# Identities of Light, Heat, and Electricity

**Published:** 1848-05

**Authors:** 


### IDENTITIES OF LIGHT, HEAT, AND ELECTRICITY.

Mr. C. Campbell Cooper has produced a tract with the above title, evincive of thought and research. After the labors of Dr. Faraday few persons, we suppose, would incline to doubt the identity of caloric, light, and electricity. The argument will be found to be presented clearly and forcibly in this pamphlet, and to be sustained by a formidable array of facts. This little work is from the press of Messrs. Grigg, Elliot & Co. We owe all the works noticed above to the politeness of Mrs. Maxwell, and Mr. F. W. Prescott, booksellers in this city.

